# Efficiency of electron cooling in cold-electron bolometers with traps

**DOI:** 10.3762/bjnano.13.80

**Published:** 2022-09-07

**Authors:** Dmitrii A Pimanov, Vladimir A Frost, Anton V Blagodatkin, Anna V Gordeeva, Andrey L Pankratov, Leonid S Kuzmin

**Affiliations:** 1 Nizhny Novgorod State Technical University, Nizhny Novgorod, Minin Street, 24, 603950, Russia; 2 Chalmers University of Technology, Department of Microtechnology and Nanoscience – MC2, Gothenburg, SE-412 96, Sweden; 3 Institute for Physics of Microstructures of the Russian Academy of Sciences, GSP-105, Nizhny Novgorod, 603950, Russia

**Keywords:** CEB, cold-electron bolometer, electron cooling, noise equivalent power, responsivity

## Abstract

Electron on-chip cooling from the base temperature of 300 mK is very important for highly sensitive detectors operating in space due to problems of dilution fridges at low gravity. Electron cooling is also important for ground-based telescopes equipped with ^3^He cryostats being able to function at any operating angle. This work is aimed at the investigation of electron cooling in the low-temperature range. New samples of cold-electron bolometers with traps and hybrid superconducting/ferromagnetic absorbers have shown a temperature reduction of the electrons in the refrigerator junctions from 300 to 82 mK, from 200 to 33 mK, and from 100 to 25 mK in the idle regime without optical power load. The electron temperature was determined by solving heat balance equations with account of the leakage current, sixth power of temperature in the whole temperature range, and the Andreev current using numerical methods and an automatic fit algorithm.

## Introduction

Cooling is a key feature to improve the sensitivity of any receiver. Reliable dilution refrigerators providing temperatures below 100 mK have not yet been implemented for operation in space under zero gravity. But ^3^He cryostats, which provide temperatures down to 250 mK, are widely used for space missions. Another advantage of ^3^He refrigerators in comparison to dilutions ones is the possibility to work at any operating angle, which is important for ground-based telescopes. Hence, it is an important task to cool down the detector as much as possible, in a different way than by just a refrigerator. One of the possible solutions of the problem is the on-chip electron cooling, which creates a drain of thermal energy from small detecting elements with the help of tunneling electrons.

Cold-electron bolometers (CEBs) [1–3] have high potential to improve the electron cooling efficiency. This concept is based on negative electrothermal feedback for an incoming signal, which is due to the direct electron cooling of the absorber by the normal metal–insulator–superconductor (NIS) tunnel junctions. Recently, in receivers with cold-electron bolometers [4–6], electron cooling from 300 to 65 mK in the idle mode without optical power load has been shown by our group [7]. Several other research groups also work in the field of electron cooling [8–13]. At present, both systems with single-stage [8–11] and double-stage [12] cooling are being used, as well as hybrid structures with graphene [13]. However, all these experiments were made without useful power load and could not be used for real experiments with detectors. The only experiments with optical power load, demonstrating background-limited operation, were carried out in [5–6,14].

Typical electron cooling in the idle mode is from 300 to 100 mK [11,15]. At low temperatures, electron cooling by a factor of 4.7 has been achieved, cooling from 150 to 32 mK [9] and from 100 to 26 mK [10]. The current record for the electron cooling factor is presented in our previous work [7]. It is 5.3 for cooling from 256 to 48 mK with an unavoidable threshold of 42 mK due to the residual Andreev current. For our measurements, new samples with CEB arrays were deposited, using the equipment of the Center for Quantum Technologies at NNSTU n.a. R.E. Alekseev. These samples have normal metal traps, as well as superconductor/ferromagnet hybrid absorbers based on Al/Fe films, as the previous samples. However, there are different oxidation parameters. This work aims to improve our new fit methodology, which takes into account both leakage and Andreev currents and also uses the sixth power of phonon and electron temperatures.

## Results and Discussion

### Experimental data fit technique

To determine the electron temperature, the contribution of the Andreev current, as well as the power of black body radiation incoming to the bolometric structure, a program in the programming language C++ has been written. It numerically solves the equations of the stationary CEB theory [16]. We use the approach based on solving the heat balance equation [7]:


[1] $$P_{\text{N}}+P_{e-\text{ph}}+2P_{\text{cool}}+2\beta P_{\text{S}}+2P_{\text{A}}+2P_{\text{leak}}=0,$$

where *P*_N_ is Joule heating in the absorber. 
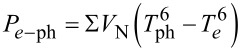
 is the heat flux between electron and phonon subsystems, taken with the sixth power [17] due to low electron temperature in our experiments (in our previous calculations we have used the fifth power). Σ is the electron–phonon coupling constant; it has different values, depending on the electron temperature [17]. *V*_N_ is the absorber volume, *P*_cool_ is the direct electron cooling power, *P*_S_ is the net power transferred to the S-electrode, and the coefficient β shows how much of *P*_S_ comes back to the absorber. *P*_A_ = *I*_A_*V* is the power due to Andreev heating current, *V* is the voltage drop across the NIS junction, and *P*_leak_ = *V*^2^/*R*_leak_ is the power associated with the leakage current.

The quasi-particle tunneling current is written as:


[2]

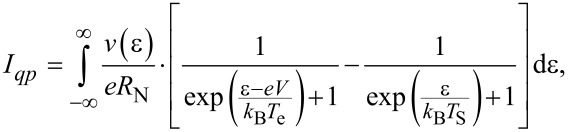



where *V* is the NIS junction voltage, *T*_e_ and *T*_s_ are the electron temperatures in the normal metal and the superconductor, 
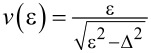
 is the density of states in the superconductor, Δ is the superconducting gap, and *k*_B_ is the Boltzmann constant.

Using the integral of the tunneling current through the NIS junction (Equation 2), the electron temperature of an absorber can be obtained [8]. This equation gives correct results if the current consists of a single-particle component only. Otherwise, we have to use a more complex approach based on Equation 1, taking both leakage and Andreev currents into account. These currents may have the same nature, since they both exist due to SN-pinholes in a tunnel barrier. Actually, it is an open question whether these currents are two different components or rather the same current but calculated with different approaches. Here we work with these two currents independently. For the planar geometry at 0 < ε < Δ, the Andreev current is expressed as [7,18]:


[3]

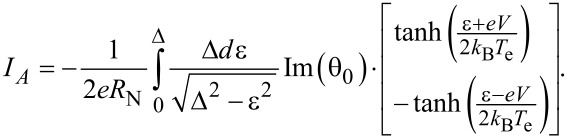



The parameterized Green's function,


[4] $$\theta_{0}=\frac{2W\Delta }{-ik^{2}\xi_{0}^{2}\sqrt{\Delta^{2}-\varepsilon^{2}}+2W\varepsilon },$$

was calculated using the Uzadel equation [19] with Kupriyanov–Lukichev boundary conditions [20], taking into account the decay of a state with a wave vector *k* due to spin scattering


[5] $$k\xi_{0}=\sqrt{\frac{\varepsilon +i/\tau_{m}}{i\Delta }}.$$

Here, τ_m_ is the magnetic scattering parameter that is found from the fit. *W* = *W*_0_ξ_0_/*d* is the effective tunneling parameter for planar tunnel junctions used in our CEB, *W*_0_ = *R*(ξ_0_)/*R*_N_ is the tunneling parameter, *R*_N_ is the normal resistance of the junction, and *R*(ξ_0_) is the resistance of Al/Fe absorber with the length ξ_0_. For aluminium, ξ_0_ = 100 nm and, for our samples, *d* = 14 nm.

Let us take a closer look at the data processing algorithm. The fit program numerically solves the equations of the stationary CEB theory (Equation 1) for a certain set of parameters and material coefficients corresponding to the measured bolometric structure. After the program run, we get the fitted current–voltage characteristics in a numerical form, as well as a set of all parameters that gives the best solution of the equations. In this way, we can determine the parameters of Andreev current and leakage current, as well as the electron temperature, to show the effectiveness of our electron cooling.

### Measurements results

**Figure 1:**
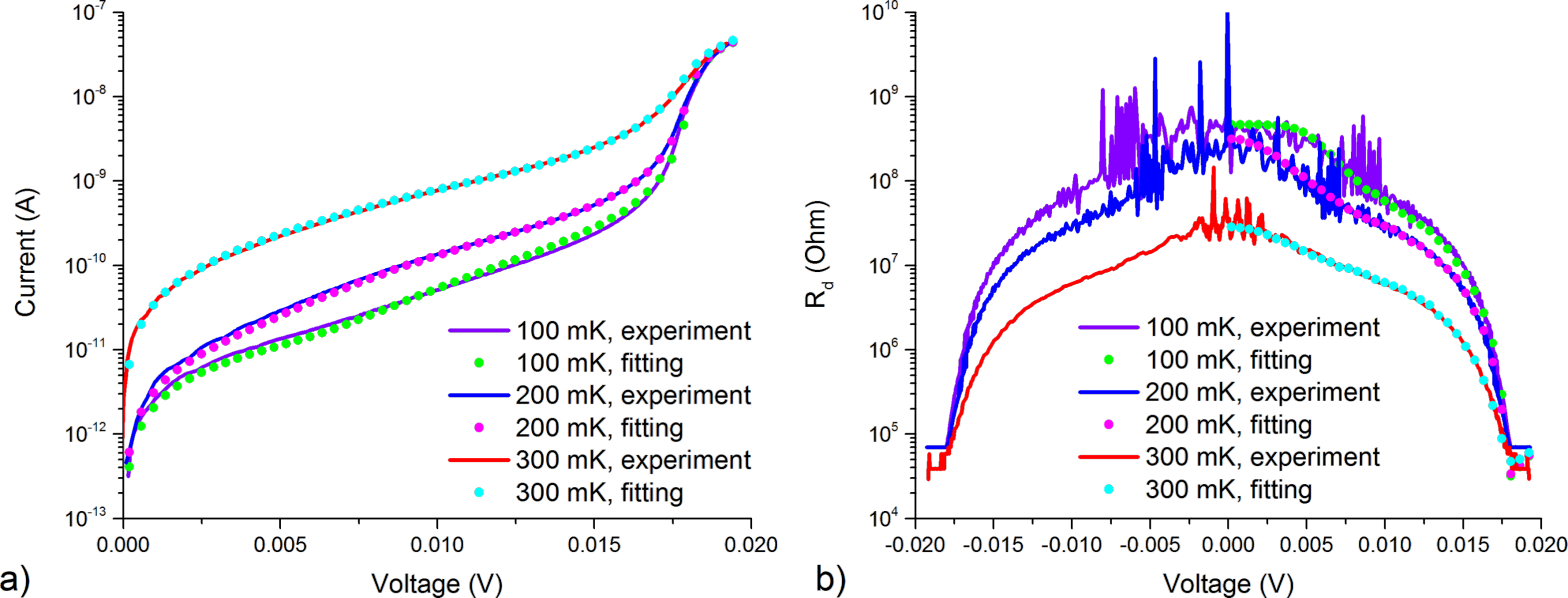
(a) Experimental current–voltage characteristics (solid curves) in comparison with theory (dots) at phonon temperatures of 300, 200, and 100 mK; (b) experimental differential resistances (solid curves) in comparison with theory (dots) at phonon temperatures of 300, 200, and 100 mK.

The sample OL-G7nn from a new sample series has the same antenna design as in [6–7,21] of a 2D array [22] with four parallel and 48 series connections, and it utilizes the same normal metal traps as in [7]. The current–voltage characteristics of this sample were measured in a Triton 200 dilution cryostat at different phonon temperatures from 100 to 300 mK. According to these characteristics, the electron temperature, as well as the contribution of Andreev and leakage currents, were determined with the use of the heat balance equation (Equation 1). The theoretical current–voltage characteristics show good matching with the experimental ones, as it can be seen in Figure 1a. In Figure 1b we show the plots of differential resistances to demonstrate that the fit agrees well not only for the current–voltage characteristics, but also for its derivatives.

**Figure 2:**
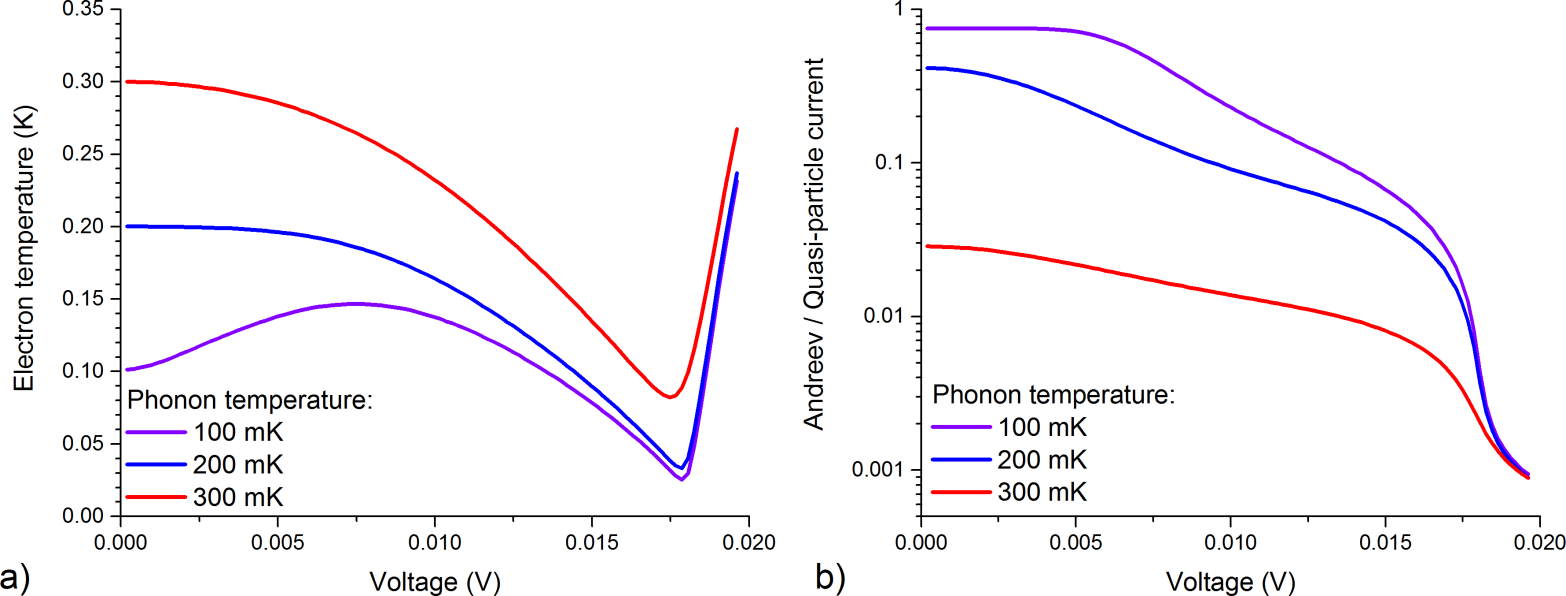
(a) The electron temperature of the absorber determined from the solution of the heat balance equation for sample OL-G7nn; (b) the ratio between Andreev current and quasi-particle current at phonon temperatures of 300, 200, and 100 mK for the same sample.

The graphs of the electron temperature of the OL-G7nn sample are shown in Figure 2a for three values of the phonon temperature of 300, 200, and 100 mK. We have started with fitting at 100 mK since Andreev and leakage currents do not change with temperature, and their contribution at lower temperatures becomes more significant, as it is seen in Figure 2b. In particular, the leakage current has been fitted with *R*_leak_ = 408 MΩ, which was determined from the differential resistance at *V* = 0 (Figure 1b), *W* and τ_m_ are 4.5 × 10^−5^ and 0.8, respectively. After that, we have managed to fit the experimental current–voltage characteristics for 200 and 300 mK with changing only the phonon temperature and Σ, which was 2.25 for 300 mK, 3.35 for 200 mK and 3.57 for 100 mK. The value of Σ depends on the electron temperature [17]. This dependence is clearly seen since the minimal electron temperatures for 100 and 200 mK are quite close, see Figure 2a.

The design of samples C from [7] and OL-G7nn is identical; the only difference is in the normal resistance due to the longer oxidation time of the OL-G7nn sample, which should lead to a thicker tunneling barrier of the NIS junctions and smaller single-particle and double-particle components of the current. For sample C, the normal resistance per one NIS junction is 1.3 kΩ, and for sample OL-G7nn this resistance is 6.4 kΩ. These differences can be seen in the electron temperature graphs: For the new sample, electron cooling is observed from 300 to 82 mK, from 200 to 33 mK, and from 100 mK to 25 mK. Therefore, cooling from a temperature of 300 mK turned out to be less efficient compared to sample C [7], for which a temperature of 65 mK was achieved. This is related to the smaller transparency of the tunnel barrier (larger resistance) and the corresponding decrease of the single-particle current, which withdraws hot electrons from the absorber. However, due to the lower Andreev heating current, which, when flowing through the normal metal absorber, leads to residual heating and, thus, restricted electron cooling, it was possible to achieve more efficient cooling in the region of low temperatures, that is, down to 25 mK (previously, for sample C, cooling only down to 42 mK was achieved).

**Figure 3:**
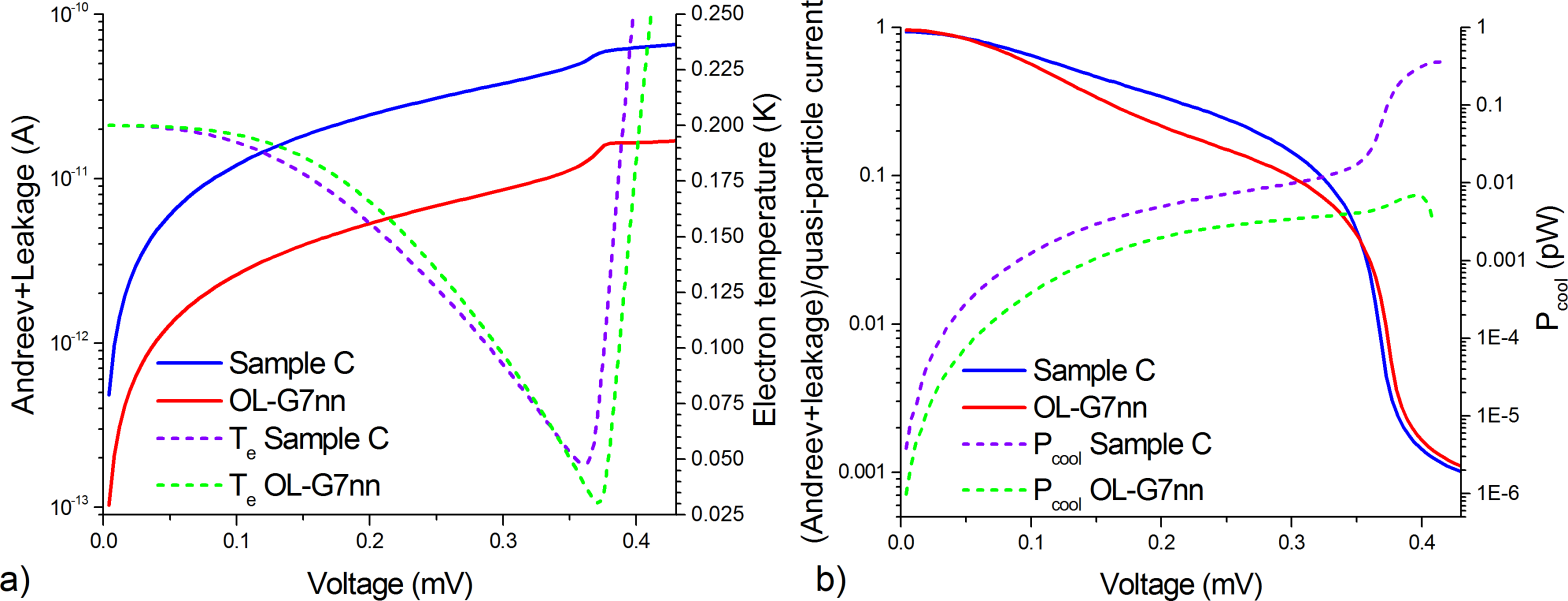
(a) The sum of the Andreev and leakage currents found by solving the heat balance equation for samples C from [7] and OL-G7nn (left axis, solid curves) at 200 mK phonon temperature, recalculated to a single bolometer in the array, and the electron temperatures for samples C and OL-G7nn (right axis, dashed curves); (b) the ratio between the sum of the Andreev and leakage currents and the quasi-particle current of two samples (left axis, solid curves), recalculated to a single bolometer in the array, and the cooling powers for samples C and OL-G7nn (right axis, dashed curves).

The comparison of the sum of the Andreev and leakage currents for sample C from [7] (blue curve) and for sample OL-G7nn (red curve) at a phonon temperature of 200 mK is presented in Figure 3a. It can be seen that, for the new sample, the Andreev and leakage currents are suppressed much stronger, which results in a lower minimal electron temperature down to 33 mK (dashed curves) at 200 mK phonon temperature. Figure 3b shows the ratio of the sum of the Andreev and leakage current components to the quasi-particle current. For the sample OL-G7nn, this sum of currents became lower with respect to the quasi-particle current. But, at the same time, the electron cooling power (dashed curves) for OL-G7nn is significantly lower, so this sample is not efficient for high background power loads of practical receivers.

Thus, in the future designs of samples, one should select parameters such that the quasi-particle current component remains rather high, but the Andreev and leakage currents are effectively suppressed due to thinner tunneling barriers with higher quality.

## Conclusion

Electron cooling is very important for highly sensitive measurements. At modern space applications, it may be the only reliable method to cool down the detector in ^3^He cryostats to achieve better sensitivity. Cold-electron bolometers are able to show electron self-cooling by a factor of five or even more [7], thus improving sensitivity, so they might be a prospective type of detectors [6].

Although we could not reach a new minimum of electron cooling at 300 mK phonon temperature, we achieved electron cooling from 200 to 33 mK and from 100 to 25 mK due to lower Andreev currents, thus decreasing our previous threshold [7] of 42 mK in the low-temperature range. For a better determination of the parameters, we have improved our fitting algorithm that takes into account both the leakage and Andreev currents and the sixth power of phonon and electron temperatures. The algorithm is able to describe the parameters of the measured sample with high accuracy, as it can be seen from comparison of experimental and theoretical current–voltage characteristics. While the studied sample demonstrates efficient cooling in the low-temperature range, it also shows drawbacks, namely smaller electron cooling power and less efficient electron cooling at 300 mK. Therefore, for practical CEB receivers operating at 300 mK at high power load, the parameters reached in [7] seem to be nearly optimal.
